# Clival Infiltrative Lesions: A Review of Differential Diagnosis Illustrated by a Rare Case of Coexisting Invasive Pituitary Adenoma and Clival Intravascular Lymphoma

**DOI:** 10.5334/jbsr.4102

**Published:** 2025-12-11

**Authors:** Eva Van Pée, Lina Daoud, Idil Gunes Tatar

**Affiliations:** 1 Department of Radiology, Cliniques Universitaires Saint-Luc, Brussels, Belgium; 2 Department of Pathology, Cliniques Universitaires Saint-Luc, Brussels, Belgium

**Keywords:** clivus, infiltrative skull base lesion, pituitary adenoma, ectopic pituitary adenoma, clival intravascular lymphoma, intravascular large B‑cell lymphoma

## Abstract

Pituitary adenoma, a common benign tumor that can invade the clivus or appear ectopically, poses diagnostic challenges. Intravascular lymphoma is extremely rare and frequently underdiagnosed. A case is presented of coexisting pituitary adenoma and clival intravascular large B‑cell lymphoma, highlighting the differential diagnosis of clival infiltrative lesions.

*Teaching point:* Clival lesions present a wide diagnostic spectrum; when imaging findings are atypical, histopathology remains essential for definitive diagnosis and appropriate treatment.

## Introduction

Clival infiltrative lesions are diagnostically challenging due to a large range of etiologies. Radiological features may be nonspecific and require histopathological confirmation.

This report illustrates the diagnostic complexity of such lesions through a case of coexisting pituitary adenoma and clival intravascular large B‑cell lymphoma (IVLBCL), and reviews the main differential diagnoses of the infiltrative clival lesions. Furthermore, the imaging and clinical features of invasive pituitary adenoma and IVLBCL are discussed.

## Case Report

**Figure 1:**
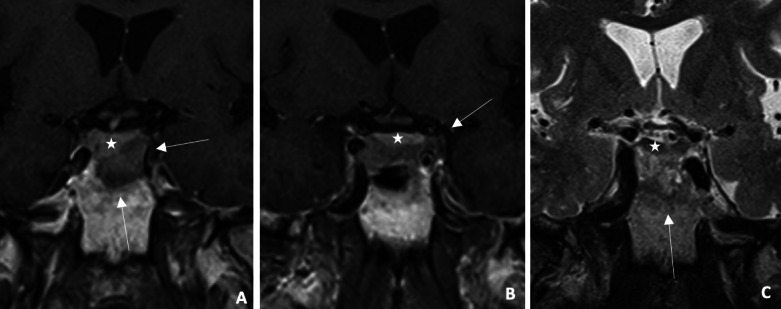
Infiltrative lesion (arrow) centered on the sellar region **(A–B)** Coronal post‑constrat T1WI: right deviation of pituitary stalk and pituitary gland (star). **(C)** Coronal T2WI: heterogeneous lesion , adjacent to hypointense pituitary gland. MRI shows infiltrative sellar lesion displacing pituitary stalk/gland.

**Figure 2:**
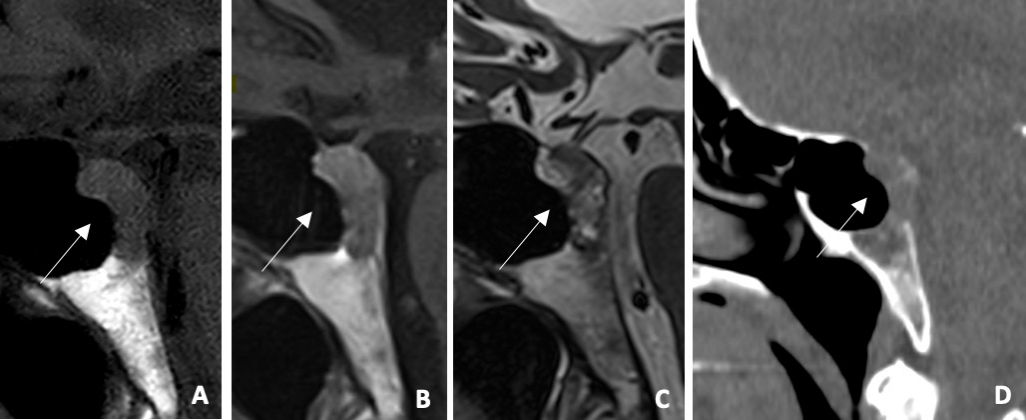
**(A)** Sagittal T1WI: hypointense expansive clival lesion **(B)** Sagittal post‑contrast T1WI: mild enhancement **(C)** Sagittal T2WI: heterogeneous lesion with hyperintense areas **(D)** Sagittal CT: Lytic clival lesion. Mildy enhancing clival lesion on MRI and lytic on CT.

**Figure 3:**
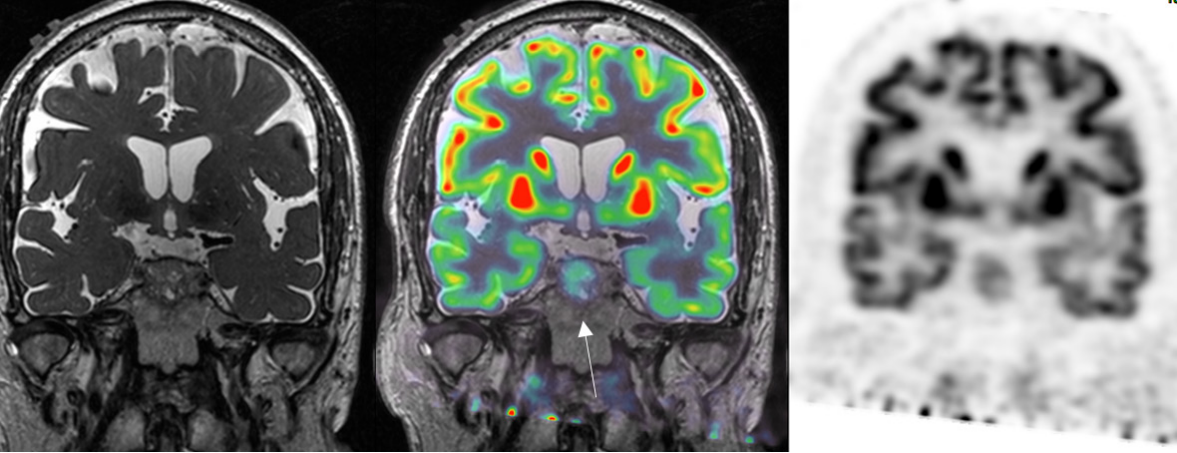
Coronal PET‑CT F18‑FDG with MRI coregistration: moderate uptake of infiltrative sellar lesion. Moderate FDG uptake of infiltrative sellar lesion.

A 77‑year‑old man with chronic balance disorder and hearing loss underwent brain magnetic resonance imaging (MRI), which revealed an infiltrative sellar lesion extending into the cavernous sinuses and clivus, with pituitary stalk/gland deviation (Figures 1 and 2). The lesion showed heterogeneous signal, with T2 hyperintense areas, differing from the hypointensity of the normal pituitary, and mild enhancement, still hypovascular compared to the gland. 18 F‑FDG PET‑CT showed a lytic lesion with moderate uptake (Figure 3).

Pituitary hormone levels were normal. Biopsy revealed both ACTH‑secreting pituitary adenoma and IVLBCL. The patient is receiving systemic chemotherapy with planned intrathecal prophylaxis.

## Discussion

### Invasive pituitary macroadenoma

These usually affect middle‑aged adults, more often women [1]. Symptoms depend on hormonal secretion or local invasion [2, 3].

Clival invasion occurs by:

1. **Embryologic origin:** During development, residual ectopic pituitary tissue may persist along the ectodermal migration path through the sphenoid bone and form an adenoma. Zhang et al. reported a case of ectopic adrenocorticotropic hormone‑secreting pituitary adenoma in the clivus [4].
2. **Anatomic extension:** As described by Wu et al., a cancellous bone corridor may allow tumor spread from the sella or the sphenoid sinus floor into the clivus and toward the occipital condyles, with potential extension to the petrous apex via the petroclival fissure [1].

On computed tomography (CT), invasive pituitary macroadenomas are characterized by bone remodeling. On MRI, they present with the same morphological characteristics as the pituitary macroadenoma: hypointense on T1‑weighted imaging (T1WI), iso‑ to hyperintense on T2WI and, in large lesions, heterogeneous signal with hyperintense areas (hemorrhagic, necrotic). They show less enhancement compared to the normal hypophysis [3].

### Intravascular large B‑cell lymphoma (IVLBCL)

IVLBCL is a rare extranodal subtype of diffuse large B‑cell lymphoma, with proliferation of malignant lymphoid cells confined to small vessels. The median age at diagnosis is about 70 years [5].

Two clinical variants exist [5, 6]: the more common Asian variant (bone marrow, spleen, liver) and the Western variant (central nervous system, skin).

Presentation is highly variable and may include systemic B symptoms, neurologic deficits, anterior pituitary hormone deficiencies, diabetes insipidus, cranial nerve dysfunction, skin lesions, lymphadenopathy, and dyspnea [6, 7]. Due to the diversity of symptoms and rarity, diagnosis is often delayed or made postmortem (53%). Definitive diagnosis requires histopathology showing proliferation of lymphoma cells within the lumina of small vessels. Treatment relies on systemic chemotherapy [6].

On MRI, IVLBCL may mimic other lesions due to its homogeneous enhancement and invasive nature. On post‑chemotherapy follow‑up imaging, tumor regression is reported [5].

### Differential diagnosis of other clival infiltrative lesions

Main differential diagnoses include chordoma, chondrosarcoma, plasmacytoma, metastasis, giant cell tumor, and nasopharyngeal carcinoma [2, 8, 9]. These can be oriented by their imaging characteristics. An overview of the typical radiologic features of the most frequent clival lesions is summarized in Table 1.

**Table 1:** Imaging‑based differential diagnosis of most frequent clival lesions.

PATHOLOGY	IMAGING FEATURES	LOCALIZATION
**Chordoma**	Hyperintense on T2WI with soap bubble appearance Thumb sign	Midline, spheno‑occipital synchondrosis
**Chondrosarcoma**	Ring or arc calcifications Hyperintense on T2WI, septa enhancement	Off‑midline, petro‑occipital synchondrosis
**Plasmacytoma**	Punched‑out lytic lesion Isointense on T1/T2WI Homogeneous enhancement	May cross synchondrosis
**Metastasis**	Variable signal, often multifocal	Anywhere
**Giant Cell Tumor**	Expansive well‑defined, cortical thinning	Sphenoïd > temporal > frontal
**Nasopharyngeal Carcinoma**	Erosive lesion, thickening of the posterior nasopharyngeal wall, affecting the adjacent soft tissues	Cavum involvement Cervical lymphadenopathy

In this case, chordoma and chondrosarcoma were excluded based on imaging characteristics, since both are typically hyperintense on T2WI, and the absence of the ‘thumb sign,’ specific to chordoma [2].

Plasmacytoma could have been considered due to overlapping MRI features: generally isointense on T1 and T2WI and homogeneous enhancement. However, it was excluded because of the absence of monoclonal B‑cell abnormalities in the blood profile and typical clinical findings such as multiple cranial nerve palsies [2, 8].

Metastasis could not be definitively excluded, but the absence of primary tumor and lack of multifocal involvement made this unlikely [2]. According to the systematic review by Jozsa et al., symptoms of clival metastasis preceded the diagnosis of the primary cancer in 43% of cases, highlighting the importance of considering metastatic origin in the differential diagnosis of clival lesions [9].

Giant cell tumor was rejected due to its rarity and patient’s age (typical age range: 20–40 years) [2].

Nasopharyngeal carcinoma was excluded by lack of cavum/nodal involvement [2].

## Conclusion

Clival lesions remain diagnostically challenging due to diverse etiologies. This case underlines the need to consider also the rare diagnosis for the selection of correct treatment. Accurate diagnosis and management require a multidisciplinary approach integrating clinical, imaging, and pathological findings.
